# Factors associated with anaemia among pregnant women in Rwanda: an analysis of the Rwanda demographic and health survey of 2020

**DOI:** 10.1186/s12884-024-06528-6

**Published:** 2024-04-27

**Authors:** Lilian Nuwabaine, Joseph Kawuki, Livingstone Kamoga, Quraish Sserwanja, Ghislaine Gatasi, Elorm Donkor, Linet M Mutisya, John Baptist Asiimwe

**Affiliations:** 1 School of Nursing and Midwifery, Aga Khan University, Kampala, Uganda; 2 Jockey Club School of Public Health and Primary Care, Faculty of Medicine, The Chinese University of Hong Kong, Hong Kong, China; 3 Department of Nursing, Makerere University College of Health Sciences, Makerere, Uganda; 4 Programmes Department, Relief International, Khartoum, Sudan; 5 Key Laboratory of Environmental Medicine Engineering, School of Public Health, Southeast University, Nanjing, 210009 Jiangsu Province China; 6 Maternal and Child Health Project, Swedish Organization for Global Health, Mayuge, Uganda

**Keywords:** Anaemia, Pregnancy, Women, Iron deficiency, Rwanda

## Abstract

**Background:**

Anaemia in pregnancy is associated with several adverse outcomes for mothers and newborns, as well as their families. In this study, we assessed the prevalence of anaemia and the associated factors among pregnant women in Rwanda.

**Methods:**

Secondary data from the 2020 Rwanda Demographic and Health Survey (RDHS) was used. Multistage stratified sampling was used to select 435 pregnant women included in the study. Anaemia among pregnant women was defined as a haemoglobin value < 11 g/dL. Multivariable logistic regression was used to assess the associated factors with anaemia in pregnancy, using SPSS (version 26).

**Results:**

Of the 435 pregnant women, 24.6% (95%CI: 21.1–29.3) were anaemic (1 in 4 pregnant women). Not working (AOR = 2.45; 95%CI: 1.14–5.26), being unmarried (AOR = 1.23; 95%CI: 1.24–3.57), low wealth index (AOR = 9.19; 95%CI: 1.64–51.56), having difficulty accessing a nearby health facility (AOR = 5.40; 95%CI: 2.21–13.23), and normal body mass index (AOR = 3.33; 95%CI: 1.46–7.59) were associated with higher odds of being anaemic. However, not taking iron supplements (AOR = 0.16; 95% CI: 0.04–0.67), having no exposure to television (AOR = 0.35; 95%CI: 0.14–0.91), being from the southern region (AOR = 0.14; 95% CI: 0.03–0.66), and low husband/partner’s education (AOR = 0.08; 95% CI: 0.01–0.59) were associated with lower odds of being anaemic.

**Conclusions:**

The study findings indicate a high prevalence of anaemia in pregnancy, which was associated with several socio-demographics. There is a need for setting up mobile clinics and health facilities in hard-to-reach areas for easy accessibility to early anaemia screening services. Conducting mass screening for anaemia targeting pregnant women who are not working, the unmarried, and those with a low wealth index would also be beneficial. The intake of locally available iron rich foods and/ or bio-fortified foods is also recommended.

## Introduction

Anaemia is a condition associated with a decrease in the mass of red blood cells or haemoglobin (Hb), limiting the blood oxygen carrying capacity [1]. Anaemia remains a public health concern and a major contributor to morbidity and mortality of pregnant women and their fetuses [2–4]. Globally, close to 2 billion people are affected by anaemia, of which 29.9% of them are women of reproductive age [5]. Currently, only one country is on the course of achieving the global target of a 50% anaemic reduction among women of reproductive age by 2025 [6].

Africa and Asia face the highest burden of anaemia (over 85%) among the high-risk groups including pregnant women [7]. In sub-Saharan Africa (SSA), pregnant women account for one-third (36.5%) of anaemia cases, with mild anaemia being the most common at 70.8% [8] and with the highest occurrence during the third trimester of pregnancy (48.8%) [9]. Several conditions predispose women to anaemia during pregnancy, such as low dietary intake of iron, folic acid, and vitamin B12, genetic causes like sickle cell disease, acute blood loss, and renal insufficiency [10, 11]. In addition, in SSA, the high incidence of anaemia during pregnancy is exacerbated by the high prevalence of infectious diseases such as malaria, Human Immunodeficiency Virus/Acquired Immunodeficiency Syndrome (HIV/AIDS), and tuberculosis, as well as hookworm infestation and schistosomiasis [2].

Anaemia during pregnancy is associated with adverse maternal and newborn health outcomes such as; increased risk of maternal and perinatal morbidity and mortality, preterm birth, intrauterine fetal death (IUFD), intrauterine growth restriction (IUGR), and low birth weight [7, 8, 12–16]. Anaemia is ranked first among the controllable and preventable causes of adverse pregnancy outcomes in SSA [17] and in developing countries, anaemia contributes to 89% of complications in pregnancy [18]. Anemia also imposes serious extra costs and implications, like a decreased workforce, impaired learning, and stunted child growth, onto society and families [9].

Multiple studies in SSA report that anaemia in pregnant women is influenced by a variety of individual, social, and contextual factors [19–22]. Residence, antenatal care (ANC) follow-up, history of excess menstrual bleeding and inter-pregnancy interval, low levels of education, and wealth index were the factors associated with anaemia among pregnant women as were reported in studies conducted in Ethiopia and some East African countries such as Uganda, and Burundi [23, 24].

The government of Rwanda has implemented several measures and strategies to improve nutrition among women of reproductive age (WRA) by promoting better maternal, infant, and young child nutrition practices, providing antenatal micronutrient supplementation, and distributing free fortified blended food supplements to pregnant women in vulnerable households [25]. In addition to the nutrition strategies, the government has also implemented measures for infection prevention and control regarding malaria in vulnerable populations by scaling up practices such as the distribution of free insecticide-treated mosquito nets and intermittent preventive therapy amongst pregnant women to control malaria which is also a cause of anaemia in this group [26]. However, despite these measures, the prevalence of anaemia among WRA stood at 13% in previous studies and was projected to be higher among pregnant women [27].

Previous studies in Rwanda have often focused on WRA in general with little mention or targeting of pregnant women [28–32]. Only one study focused on anaemia among pregnant women [33]. Moreover, this was a hospital-based study, and therefore not representative of all pregnant women in Rwanda [33]. Another study determining the prevalence of anaemia among pregnant women was on a wider East African region not necessarily focusing on one country (Rwanda) and analyzed old data from 2014 [34]. Given the most recent national efforts aimed at addressing anaemia, a more-recent analysis of Rwandan progress was needed. Therefore, this study sought to determine the prevalence of anaemia and associated factors among pregnant women in Rwanda, using a nationwide dataset of the 2020 Demographic and Health survey. Understanding the associated factors related to anaemia in pregnancy may be vital in guiding policy and implementing targeted measures to accelerate progress toward controlling this public health problem.

## Methods

### Study sampling and participants

This analysis used data from the 2019-20 Rwanda Demographic and Health Survey (RDHS) that was collected from November 2019 to July 2020. The cross-sectional study used a two-stage sample design; the first stage involved cluster selection consisting of enumeration areas (EAs), and the second stage involved systematic sampling of households in all the selected EAs, leading to a total of 13,005 households [27]. In particular, the data used in this analysis were from the household and the women’s questionnaires.

Women of reproductive age (15–49 years) who were either permanent residents of the selected households or visitors who stayed in the household the night before the survey were eligible for interview. Out of the total 13,005 households that were selected for the survey, 12,951 were occupied and 12,949 were successfully interviewed leading to a 99.9% response rate [27]. This analysis included only the interviewed pregnant women whose number was 870 out of the total 14,634 women in the whole survey. However, of the eligible 870 pregnant women, 435 had missing values for Haemoglobin levels, as they did not agree to take the anaemia test- and so these were excluded, giving us a valid sample of 435 pregnant women used in this analysis [27]. In other words, we used a sample that agreed to take the anaemia test during the survey. Details of specific procedures of RDHS anaemia tests are published elsewhere [27].

### Variables

#### Dependent variables

This study was based on altitude-adjusted haemoglobin levels, which were already provided in the DHS dataset [35]. The outcome variable was anaemia level considered as a haemoglobin value < 11 g/dl and was re-categorized as a binary variable (anaemic coded as yes = 1 and non-anaemic coded as no = 0). Anaemia cases in RDHS dataset were further categorised as mild (10.0–10.9 g/dl), moderate (7.0–9.9 g/dl) and severe (< 7.0 g/dl) anaemia [27].

### Explanatory variables

We included possible determinants of anaemia based on the available literature and data [23, 35, 36]. Twenty-five (25) variables were considered and of these, three were community factors that included; place of residence (categorized into rural and urban), region of residence (Kigali, South, West, East, and North), and perceived problem with distance to a health facility (big problem and no big problem). Six household-level factors included; household size (classified into “less than six” and “six and above”), sex of household head (male and female), husband/partner’s educational level, source of drinking water (improved and unimproved), type of toilet facility (improved and unimproved) and wealth index (categorized into five quintiles that ranged from the poorest to the richest quintile). Individual-level factors were also considered in the analysis and included; age (categorized as 15–24, 25–34, 35–44, and 45–49 years), working status (yes or no), parity (4 and less and above 4), educational level (no education, primary, secondary and tertiary), marital status (married and unmarried), pregnancy trimester (first, second and third), deworming (yes and no), mosquito net use (yes and no), health insurance (yes and no), body mass index (underweight, normal, overweight and obese), visited a health facility in the last 6 months (yes and no), antenatal care visits (below 4 or 4 and above), iron supplementation during pregnancy (yes or no), and exposure to radio, newspapers, and television (yes and no). The wealth index was calculated by RDHS from information on household asset ownership using Principal Component Analysis. To assess iron supplementation during pregnancy, DHS asked mothers “During pregnancy, were you given or did you buy iron tablets or syrup” and “Days the tablet or syrup was taken”. For our analysis, only the former question was used [27].

### Statistical analysis

We applied the DHS sample weights to account for the unequal probability sampling in different strata and ensure the representativeness of the study results [37, 38]. We used SPSS (version 26.0) software - complex samples package, incorporating the following variables in the analysis plan to account for the multistage sample design inherent in the RDHS dataset: individual sample weight, sample strata for sampling errors/design, and cluster number [27, 37, 39]. Frequency distributions were used to describe the background characteristics of the respondents, where frequencies and proportions/percentages for categorical dependent and independent variables were presented. The chi-square test was used to assess for differences in the distribution of background variables by anaemic status. Simple logistic regression was then conducted to assess the independent association of each predictor variable with anaemia. Crude odds ratio (COR), 95% confidence interval (CI), and *p*-values are presented. Independent variables found significant at a *p*-value less than 0.25 were then included in the multivariable model. Moreover, independent variables that were non-significant at the bivariable analysis level but were associated with anaemia in previous studies were also included in the multivariable logistic regression model. In other words, we executed an “all-inclusive” model. We used the backward-elimination method during step-wise regression, by discarding the least statistically significant variable until we remained with only significant variables. At each elimination step, the results of the discarded variable were recorded. Adjusted odds ratios (AOR), 95%CI, and *p*-values were obtained and presented, with a statistical significance level set at *p*-value < 0.05. All socio-demographic variables in the model were assessed for multi-collinearity, which was considered present if the variables had a variance inflation factor (VIF) greater than 10 [38]. However, none of the variables had a VIF above 3.

## Results

### Characteristics of participants

A total of 435 pregnant women were included in this analysis. The majority (71.8%) were below 35 years of age, married (85.2%), with a primary level of education (62.3%), working (65.5%), and with parity of below 4 (81.7%). Most of the respondents had health insurance (86.7%), resided in rural areas (83.1%), were from male-headed households (80.4%) of less than 6 members (77.4%), and had exposure to the radio (79.4%) but not newspapers (79.6%). In addition, 55.2% had normal body weight, 58.5% used mosquito nets, 45.2% had been dewormed, 83.7% had visited a health facility in the past 6 months and 73.7% had no big problem with the distance to health facility (Table 1). Of the analysed sample, 107 (24.6%, 95%CI: 21.1–29.3) pregnant women had anaemia, where 71(16.3%), 35(8.1%) and 1(0.2%) had mild, moderate and severe anaemia respectively. There was no big difference in background characteristics by anaemia status (anaemia vs. no anaemia) apart from working status, health insurance, husband’s education level, pregnancy trimester, and having problems with distance to a health facility (Table 1).

**Table 1:** Background characteristics of pregnant women as per the 2020 Rwanda Demographic Health Survey

Characteristics	Overall Frequency *n (%)*, *N* = 435	Anaemia *n (%)*, *N* = 107	No anaemia *n (%)*, *N* = 328	*P*-value
**Age**				0.317
15–24	119(27.4)	30(28.0)	89(27.1)	
25–34	193(44.4)	41(38.3)	152(46.3)	
35 and above	122(28.2)	36(33.6)	87(26.5)	
**Education level**				0.916
Tertiary	19(4.4)	5(4.7)	14(4.3)	
Secondary	113(26.0)	26(24.3)	87(26.5)	
Primary	271(62.3)	66(61.7)	205(62.5)	
No education	32(7.3)	10(9.3)	22(6.7)	
**Working status**				**0.018**
Working	285(65.5)	60(56.1)	225(68.6)	
Not working	150(34.5)	47(43.9)	103(31.4)	
**Parity**				0.334
Below 4	355(81.7)	91(85.0)	264(80.5)	
4 and above	80(18.3)	16(15.0)	64(19.5)	
**Marital status**				0.181
Married	370(85.2)	87(81.3)	284(86.6)	
Unmarried	64(14.8)	20(18.7)	44(13.4)	
**Health insurance**				**0.049**
Yes	377(86.7)	99(92.5)	279(84.8)	
No	58(13.3)	8(7.5)	50(15.2)	
**Wealth index**				0.984
Richest	90(20.8)	22(20.6)	69(21.0)	
Richer	89(20.5)	24(22.4)	65(19.8)	
Middle	89(20.5)	21(19.6)	68(20.7)	
Poorer	85(19.5)	20(18.7)	65(19.8)	
Poorest	81(18.7)	20(18.7)	61(18.6)	
**Residence**				0.517
Urban	73(16.8)	20(18.9)	53(16.2)	
Rural	362(83.2)	87(81.1)	275(83.8)	
**Region**				0.334
Kigali	56(12.9)	18(16.8)	38(11.6)	
West	103(23.7)	26(24.3)	77(23.5)	
East	103(23.7)	24(22.2)	79(24.0)	
North	64(14.7)	18(16.8)	46(14.0)	
South	109(25.0)	21(19.6)	88(26.8)	
**Household size**				0.187
Less than 6	336(77.2)	88(81.3)	249(75.9)	
6 and above	99(22.8)	20(18.7)	79(24.1)	
**Sex of household head**				0.528
Male	349(80.2)	84(77.6)	266(81.1)	
Female	86(19.8)	24(22.4)	62(18.9)	
**Exposure to radio**				0.432
Yes	345(79.4)	82(76.6)	263(80.2)	
No	89(20.6)	25(23.4)	65(19.8)	
**Exposure to television**				0.171
Yes	176(40.5)	49(45.8)	127(38.7)	
No	259(59.5)	58(54.2)	201(61.3)	
**Exposure to newspapers**				0.486
Yes	89(20.4)	25(23.4)	64(19.5)	
No	346(79.6)	82(76.6)	264(80.5)	
**Husband/partner’s education** ^**a**^				**0.023**
Tertiary	27(6.2)	12(13.6)	15(5.3)	
Secondary	61(14.0)	18(20.5)	43(15.2)	
Primary	236(54.3)	49(55.6)	188(66.4)	
No education	47(10.8)	10(11.4)	37(13.1)	
**Mosquito net use**				0.607
Yes	254(58.5)	65(60.7)	190(57.9)	
No	180(41.5)	42(39.3)	138(42.1)	
**Deworming** ^**b**^				0.839
Yes	196(45.2)	44(86.3)	152(87.4)	
No	29(6.5)	7(13.7)	22(12.6)	
**Trimester**				**0.029**
First	138(31.7)	24(22.4)	114(34.8)	
Second	138(31.8)	43(40.2)	95(29.0)	
Third	159(36.5)	40(37.4)	119(36.3	
**Problem with distance to a health facility**				**0.010**
No big problem	320(73.6)	68(63.6)	252(76.8)	
Big problem	115(26.4)	39(36.4)	76(23.2)	
**Visited health facility in last 6 months**				0.417
Yes	363(83.5)	92(86.0)	271(82.6)	
No	72(16.5)	15(14.0)	57(17.4)	
**Body mass index (BMI)**				0.093
Overweight	187(43.0)	37(34.6)	150(45.7)	
Normal	240(55.2)	69(64.5)	171(52.1)	
Underweight	8(1.8)	1(0.9)	7(2.1)	
**Antenatal care visits** ^**c**^				0.164
Below 4	116(49.8)	21(40.4)	95(52.2)	
4 and above	118(50.2)	31(59.6)	87(47.8)	
**Iron supplementation taken** ^**c**^				0.011
No	47(19.9)	4(7.7)	43(23.6)	
Yes	187(80.1)	48(92.3)	139(76.4)	
**Source of drinking water**				0.966
Improved source	349(80.3)	86(80.4)	263(80.2)	
Unimproved source	86(19.7)	21(19.6)	65(19.8)	
**Type of toilet facility**				0.327
Improved facility	309(71.1)	80(74.8)	229(69.8)	
Unimproved facility	127(28.9)	27(25.2)	99(30.2)	
**Anaemic**				
No	328(75.4)			
Yes	**107(24.6), (95%CI: 21.1–29.3)**			

a, b and c had many missing values.

### Factors associated with anaemia in pregnancy

Results of the bivariable analysis with their respective crude odds ratios and *p*-values are detailed in Table 2. At multivariable analysis controlling for all the included variables, the factors found significantly associated with anaemia were; working status, marital status, wealth index, the problem with distance to the health facility, body mass index, region of residence, exposure to television, husband/partner’s education and iron supplementation taken during pregnancy.

Compared with working pregnant women, those not working had 2.45 (95%CI: 1.14–5.26) higher odds of being anaemic, while the unmarried had 1.23 (95%CI: 1.24–3.57) higher odds of being anaemic compared with married counterparts. Pregnant women in the richer (AOR = 5.79, 95%CI: 1.46–22.96) and middle (AOR = 9.19, 95%CI:1.64–51.56) wealth quintiles had higher odds of being anaemic compared with their counterparts in the richest wealth quintile. In addition, pregnant women having a big problem with distance to a health facility (AOR = 5.40, 95%CI: 2.21–13.23) and those of normal body mass index (AOR = 3.33, 95%CI: 1.46–7.59) also had higher odds of being anaemic, compared with their counterparts with no big problem and overweight BMI respectively.

However, pregnant women who did not take iron supplementation (AOR = 0.16, 95%CI: 0.04–0.67), those with no exposure to television (AOR = 0.35, 95%CI: 0.14–0.91), and those residing in the southern region (AOR = 0.14, 95%CI: 0.03–0.66) were 84%, 65% and 86% less likely to be anaemic, compared with their respective counterparts who took iron supplements, were exposed to television, and those who lived in Kigali region. Similarly, pregnant women with husbands of primary (AOR = 0.08, 95%CI: 0.01–0.50) and no education (AOR = 0.08, 95%CI: 0.01–0.59) were both 92%, less likely to be anaemic compared with those with husbands of tertiary education (Table 2).

**Table 2:** Factors associated with anaemia among pregnant women as per the 2020 Rwanda Demographic Health Survey

Variable	Crude odds ratio, COR (95%CI)	*p*-value*	Adjusted odds ratio, AOR (95%CI)	*p*-value**
**Age**		0.381		0.938
15–24	1		1	
25–34	0.81(0.44–1.51)		1.26(0.27–5.93)	
35 and above	1.20(0.64–2.26)		1.37(0.23–8.27)	
**Education level**		0.891		0.168
Tertiary	1		1	
Secondary	0.80(0.25–2.59)		0.53(0.06–4.74)	
Primary	0.89(0.29–2.72)		0.30(0.03–2.99)	
No education	1.16(0.32–4.19)		1.05(0.07–16.45)	
**Working status**		**0.030**		**0.022**
Working	1		1	
Not working	1.71(1.05–2.79)		**2.45(1.14–5.26)**	
**Parity**		0786		0.960
Below 4	1		1	
4 and above	0.88(0.49–1.60)		0.95(0.13–7.01)	
**Marital status**		**0.240**		**0.039**
Married	1		1	
Unmarried	1.47(0.77–2.78)		**1.23(1.24–3.57)**	
**Health insurance**		**0.061**		0.197
Yes	1		1	
No	0.47(0.21–1.04)		0.32(0.06–1.82)	
**Wealth index**		0.986		**0.029**
Richest	1		1	
Richer	1.14(0.54–2.40)		**5.79(1.46–22.96)**	
Middle	0.98(0.45–2.13)		**9.19(1.64–51.56)**	
Poorer	0.94(0.44–2.03)		4.20(0.76–23.07)	
Poorest	1.05(0.48–2.28)		4.28(0.84–21.92)	
**Residence**		0.578		0.794
Urban	1		1	
Rural	0.83(0.43–1.60)		1.16(0.37–3.65)	
**Region**		0.497		**0.042**
Kigali	1		1	
West	0.72(0.30–1.74)		0.30(0.07–1.29)	
East	0.66(0.28–1.57)		0.21(0.04–1.04)	
North	0.84(0.32–2.18)		0.39(0.07–2.28)	
South	0.48(0.20–1.16)		**0.14(0.03–0.66)**	
**Household size**		0.280		0.542
Less than 6	1		1	
6 and above	0.70(0.37–1.33)		1.43(0.45–4.55)	
**Sex of household head**		0.545		0.261
Male	1		1	
Female	1.19(0.67–2.12)		0.54(0.18–1.60)	
**Exposure to radio**		0.480		0.576
Yes	1		1	
No	1.22(0.70–2.13)		1.48(0.37–5.85)	
**Exposure to television**		**0.195**		**0.032**
Yes	1		1	
No	0.73(0.45–1.18)		**0.35(0.14–0.91)**	
**Exposure to newspapers**		0.518		0.463
Yes	1		1	
No	0.82(0.45–1.50)		0.64(0.19–2.12)	
**Husband/partner’s education**		**0.102**		**0.032**
Tertiary	1		1	
Secondary	0.53(0.18–1.62)		0.11(0.01–1.83)	
Primary	0.33(0.12–0.89)		**0.08(0.01–0.50)**	
No education	0.36(0.11–1.13)		**0.08(0.01–0.59)**	
**Mosquito net use**		0.608		0.777
Yes	1		1	
No	0.88(0.55–1.42)		0.86(0.30–2.49)	
**Deworming**		0.895		0.384
Yes	1		1	
No	1.07(0.367–3.148)		1.75(0.49–6.23)	
**Trimester**		**0.066**		0.891
First	1		1	
Second	2.11(1.13–3.93)		0.81(0.25–2.56)	
Third	1.59(0.86–2.93)		0.74(0.21–2.58)	
**Problem with distance to a health facility**		**0.023**		**<0.001**
No big problem	1		1	
Big problem	1.84(1.09–3.12)		**5.40(2.21–13.23)**	
**Visited health facility in last 6 months**		0.479		0.255
Yes	1		1	
No	0.77(0.37–1.59)		1.88(0.63–5.63)	
**Body mass index (BMI)**		**0.120**		**0.014**
Overweight	1		1	
Normal	1.65(1.01–2.72)		**3.33(1.46–7.59)**	
Underweight	0.77(0.08–7.04)		8.05(0.77–83.78)	
**Antenatal care visits**		**0.221**		0.962
4 and above	1		1	
Below 4	0.65(0.33–1.30)		0.98(0.37–2.58)	
**Iron supplementation taken during pregnancy**		**0.012**		**0.013**
Yes	1		1	
No	0.24(0.08–0.73)		**0.16(0.04–0.67)**	
**Source of drinking water**		0.947		0.606
Improved source	1		1	
Unimproved source	0.98(0.55–1.74)		0.74(0.24–2.32)	
**Type of toilet facility**		0.345		0.235
Improved facility	1		1	
Unimproved facility	0.78(0.47–1.31)		0.52(0.18–1.54)	

**Bold** = significant, *= significant at 0.25, **= significant at 0.05, CI = confidence interval.

## Discussion

Given the insidious nature of anaemia and its adverse effects, we assessed its prevalence and associated factors among pregnant women in Rwanda. Although we found a 24.6% prevalence of anaemia among pregnant women, which is lower than the East African, and the global prevalence of anaemia in pregnant women reported at 41.82% and 39.1% respectively [34, 40], anaemia remains a huge problem within Rwanda which requires redress. The discrepancies in the prevalence of anaemia reported in the various studies may be attributed to the differences in nutritional behaviours and culture of women in the various settings, such as the high consumption of folate and iron-rich foods (green leafy vegetables) in many cultures across Africa [41]. The observed high prevalence of anaemia in this study may also indicate a significant nutritional problem among pregnant women in Rwanda despite the several efforts such as policies on food bio-fortification that have been put in place to reduce and control anaemia in the country [38]. This may imply that there is limited adoption, or implementation of the food biofortification policy and /or low awareness, availability, and consumption of iron bio-fortified foods (such as beans and orange fleshed sweat potatoes) in homes by mothers during the preconception and pregnancy stages [42].

In this study, various factors were found to be significantly associated with anaemia. Pregnant women who were not working were almost twice more likely to be anaemic than those who were working. This is similar to findings from studies conducted in East Africa and Nigeria which found a positive impact of working status on anaemia [33, 39, 40]. This may be partly because pregnant women who are working are more likely to be in contact with various women (social networks) who may provide nutritional advice during pregnancy and/ or visit health facilities where they obtain advice and iron-rich tablets unlike those who are not working.

Pregnant women from richer households had higher odds of being anaemic compared with those from the richest wealth index. This may be because coming from wealthier households could also mean that women also access better health services and healthier, sufficient food than their counterparts with less income. This agrees with results from several studies conducted in Tanzania, Sri Lanka, Nigeria, Rwanda, Uganda, and Ethiopia which found women with low wealth index being more likely to be anaemic [28, 43–47]. Moreover, although our observation generally agrees with other studies that anaemia risk increases with decreasing wealth index, it is quite surprising it was the rich quintile that was significant not the poorer nor poorest quintile. The higher anaemia risk even in rich women, despite the food affordability, may be explained by other factors such as negative perceptions about taking iron supplements during pregnancy, and poor food choices such as overindulging in unhealthy foods [48, 49]. This implies customised health education for wealthier women highlighting the possible risks and focusing on the possible causes of anaemia in this group, where affordability of nutritious foods may not be a concern, is needed.

In this study, we found that unmarried pregnant women were more likely to have anaemia compared with their counterparts who were married. This is in agreement with studies conducted in Malaysia and Nigeria which also found that unmarried women had higher odds of anaemia [50, 51]. On the contrary, a study conducted in Rwanda found that anaemia was more likely to occur among married women than unmarried [28]. This may be because traditionally in SSA, men are the household providers who ensure food security at their homes thus the availability of nutrient-rich foods to their wives. In Rwanda, female-headed households are more vulnerable and often have lower socioeconomic status making them less likely to have access to healthcare [52]. Additionally, married women may have benefited from spousal financial support which unmarried women may not have, and are therefore able to afford a more diverse and nutrient-rich diet.

Furthermore, we found that pregnant women who had not taken iron supplements during their current pregnancy were 84% less likely to be anaemic, compared with their respective counterparts who took iron supplements. Although there was no accessible comparable study for this finding, it is contrary to what is expected. Evidence indicates that iron supplementation in pregnancy reduces anemia [53, 54]. This inconsistency in this study finding may be because such women may have knowledge, consistent access and adherence to alternative iron-rich foods compared with their counterparts who opted to use iron supplements. Thus, mass media campaigns may need to emphasize the consumption of locally available iron-rich foods as alternatives.

The participant’s region of origin was also associated with anaemia in pregnancy, whereby women from the southern region were less likely to be anaemic compared with those living in Kigali region which is the most food-secure region in Rwanda [55]. The finding is inconsistent with previous studies which reported more anaemia cases among women in the southern region of Rwanda [28, 29]. Further research may need to be conducted to explore such discrepancies within the same region. However, one plausible explanation that may need to be further explored is the difference in iron-rich food intake, parasitic infections (hookworm infestations), and malaria infestations across the regions.

Pregnant women whose husbands/partners had primary or no formal education were less likely to be anaemic in this study. On the contrary, studies conducted in Nepal, India, and sub-Saharan Africa [56–58] found that women whose husbands/partners had a higher education level were less likely to be affected by anaemia during pregnancy. The observed pattern, however, may be due to misinformation and perceptions about taking iron supplements during pregnancy, especially among the educated. Therefore, nutritional sensitization campaigns should also involve the elites or educated men, to solicit for spousal support in the prevention of anaemia [59].

Our study found that women without Television access were 65% less likely to be anaemic, while other studies found that no Television exposure increased anaemia risk in children and adolescents [60, 61]. Interestingly, a study in Lao showed that frequent Television viewing was associated with a lower risk of anaemia in women of reproductive age [62]. This inconsistency in our study finding may indicate the provision of limited and or ineffective anemia control messages on mass media. Thus, messages such as those related to iron supplementation across mass media (such as televisions) may need to be improved. Above all, more research needs to be done further to explain the complex relationship between anemia and television exposure and to determine the presence of other causal factors.

Pregnant women having a big problem with distance to a health facility had higher odds of being anaemic compared with those with no big problem. The finding is similar to a study conducted in Eastern Africa which also reported a negative impact of distance to a health facility on the anaemia status of women [35]. This is partly because longer distance, which comes with time loss and financial implications in terms of transport, hinders access to the needed health care from health facilities. As a result, they are more likely to miss out on being screened for anaemia, health nutritional education, and iron supplements during antenatal care. To make accessibility easy for such women from hard-to-reach areas, additional services such as the provision of antenatal care including anaemia screening and supply of iron supplements should be extended to those communities through outreach programs.

We noted in this study that women with normal body mass index (BMI) were more likely to be anaemic compared with the overweight. The finding is contrary to previous studies that indicate overweight to be a predictor of micronutrient deficiency in pregnancy [63] but concurs with a study that found that an increase in BMI is associated with a reduction in the risk of anaemia in pregnancy [64]. Nonetheless, given the physiological changes and weight gain in pregnancy, this study finding implies that more specific anaemia screening indicators should be used not just BMI in pregnancy.

### Strengths and limitations

We used weighted data from the Rwanda Demographic Health survey, which is a national representative, hence these results obtained can be generalized to all pregnant women of Rwanda. In addition, DHS are known to have high response rates, with quality standardized data collection procedures across countries, thus our findings can be compared with other studies of the same design elsewhere. However, because this study used a cross-sectional design, the findings may be weak in terms of conferring causality, and there could be a risk for a recall bias from the participants since this was self-reported data. There was also a lack of data on other explanatory variables associated with anaemia, such as parasitic and malaria infection. Also, a small sample size was another limitation of this study. Specifically, half of the eligible women were excluded due to missing values of the outcome variable, and this might affect our inference of the true prevalence of the outcome variable (anaemia). Despite those limitations, the study provides valuable information on the factors associated with anaemia among pregnant women in Rwanda.

## Conclusions

The prevalence of anaemia among pregnant women was found to be high in this study (1 in 4 pregnant women were anaemic). The study unveiled working status, marital status, wealth index, body mass index, distance from the health facility, no exposure to television, lack of iron supplementation in pregnancy, and region of residence as the factors significantly associated with anaemia among pregnant women. We, therefore, recommend setting up health facilities and mobile clinics as well as strengthening community outreach programs in hard-to-reach areas to enable accessibility to early screening services. In addition, scaling up the growing of iron-rich and bio-fortified foods across regions, as well as involving men in all activities which aim at improving women’s health would be vital strategies in successful anaemia control programs. It is imperative that communication about iron supplementation across mass media (such as televisions) may need to be improved and the intake of locally available iron-rich foods as alternatives may need to be emphasized.

## Data Availability

The data set used is openly available upon permission from the MEASURE DHS website (URL: https://www.dhsprogram.com/data/available-datasets.cfm). However, authors are not authorized to share this data set with the public but anyone interested in the data set can seek it with written permission from the MEASURE DHS website (URL: https://www.dhsprogram.com/data/available-datasets.cfm).

## Abbreviations

ANC: Antenatal Care
IUGR: Intrauterine Growth Restriction
IUFD: Intrauterine Fetal Death
HIV: Human Immunodeficiency Syndrome
AIDS: Acquired Immunodeficiency Syndrome
EAL: Enumeration area
SSA: Sub Saharan Africa
AOR: Adjusted Odds Ratio
RDHS: Rwanda Demographic Health Survey
CI: Confidence Interval
DHS: Demographic Health Survey
VIF: Variance Inflation Factor
COR: Crude Odds Ratio
OR: Odds Ratio
SPSS: Statistical Package for Social Science
